# Association of lipid markers with coronary heart disease and stroke mortality: A 15-year follow-up study 

**DOI:** 10.22038/ijbms.2019.35617.8775

**Published:** 2019-11

**Authors:** Bagher Pahlavanzade, Farid Zayeri, Taban Baghfalaki, Omid Mozafari, Davood Khalili, Fereidoun Azizi, Alireza Abadi

**Affiliations:** 1 Departments of Biostatistics, Faculty of Paramedical Sciences, Shahid Beheshti University of Medical Sciences, Tehran, Iran; 2 Departments of Statistics, Faculty of Mathematical Sciences, Tarbiat Modares University, Tehran, Iran; 3 Golestan University of Medical Sciences, Gorgan, Iran; 4 Prevention of Metabolic Disorders Research Center, Research Institute for Endocrine Sciences, Shahid Beheshti University of Medical Sciences, Tehran, Iran; 5 Endocrine Research Center, Research Institute for Endocrine Sciences, Shahid Beheshti University of Medical Sciences, Tehran, Iran; 6 Department of Community Medicine, Faculty of Medicine, Shahid Beheshti University of Medical Sciences, Tehran, Iran

**Keywords:** Coronary heart disease, Cholesterol HDL, Cholesterol LDL, Stroke, Survival analysis

## Abstract

**Objective(s):**

It has been proposed that lipid markers may predict cardiovascular events; however, their effect may vary depending on the type of cardiovascular disease. The purpose of this study was to investigate the effects of lipid markers on death from coronary heart disease (CHD) and stroke in competing risks setting.

**Materials and Methods:**

Participants included 2502 women and 2020 men, age 40 years or older from Tehran Lipid and Glucose Study. The association between total cholesterol (TC), low-density lipoprotein cholesterol (LDL-C), triglyceride (TG), and high-density lipoprotein cholesterol (HDL-C) with hazard and cumulative incidence of CHD and stroke was investigated using cause-specific hazard and sub-distribution hazard models. Statistical analyses were performed using “risk regression” and “cmprsk” package in R 3.3.2.

**Results:**

One standard deviation (SD) increase in TC and LDL-C increased the hazard of CHD death by 1.42 (CI=1.07,1.89) and 1.41 (CI=1.04,1.93), respectively. 1-SD increase in TG increased the cumulative incidence of CHD death increased by 1.94 (CI=1.02,3.75) in women. Other risk factors were not associated with the hazard and cumulative incidence of CHD in women, men and the total sample. In addition, none of lipids had a significant effect on the hazard and cumulative incidence of stroke in men, women and the total sample.

**Conclusion:**

The associations of lipid components on CHD death were modiﬁed by gender. TC, LDL-C and TG were independent predictors of CHD mortality in women. Furthermore, death due to stroke changes the association of lipid markers with CHD mortality.

## Introduction

Cardiovascular diseases (CVDs) claim 17.9 million lives every year, accounting for 31% of all deaths globally. Over 75% of CVD-related deaths take place in low- and middle-income countries (1). There are six different types of CVDs (2). About 85% of all CVD deaths are due to CHD and stroke (1). In Iran, 43% of all mortalities were attributed to CVD (3), from which 25.2% were due to Coronary Heart Disease (CHD) and 14.2% due to stroke (4).

Despite their similar pathophysiology, different risk factors have been reported for these events (5-8). An association between total cholesterol (TC), low-density lipoprotein cholesterol (LDL-C), high-density lipoprotein cholesterol (HDL-C), and triglycerides (TG) with CHD-related deaths has been reported in some prospective studies (9, 10), but not in others (11, 12). A high level of TC, LDL-C, TC and a low level of HDL-C were found as risk factors of CHD (13). However, research on the association of each cholesterol sub-fraction with stroke has shown inconsistent results (2, 14, 15). Similar inconsistencies have been observed on the association of lipid markers and deaths due to CHD.

Depending on the purpose of the competing risk study, cause-specific hazard model or sub-distribution hazard model has been used for analysis. Although many studies have investigated the effect of lipid markers under cause-specific hazard model (7, 8, 16-18), studies under sub-distribution hazard (Fine and Gray) model are rare (10, 11, 19, 20). In addition, most of the studies under sub-distribution hazard had considered CVD or CHD versus all other deaths as two competing risks. CHD and stroke have been considered as two competing risks in cause-specific hazard model (7, 16-18) and sub-distribution hazard model (20).

When there are competing events, utilizing both cause-specific and sub-distribution hazard models has been recommended (21). Also in some of the previous studies (10, 11, 19) both fine and gray model and cox proportional hazard model have been utilized. These studies investigated the risk factors of CHD (CAD) versus other causes of death. In the present study, we investigate the association between TC, LDL-C, HDL-C, and TG with hazard of death from CHD and stroke and also with cumulative incidence of CHD and stroke mortality.

## Materials and Methods

***Study population***

This study is part of the Tehran Lipid and Glucose Study (TLGS), a population-based cohort study initiated in 1999-2001 and consisting of 15,010 residents (age > 3 years) of 13 districts of the city of Tehran, Iran (22). Samples were selected through multi-stage stratified cluster random sampling (22).

Initially 5089 participants, age ≥ 40 years at baseline (1999–2001), were selected for the current study. From this group, 611 individuals died during the 15 years of follow-up (53 from stroke, 63 from CHD, and 495 from other causes). Furthermore, subjects with missing data on lipid markers (567) were excluded. The study protocol was approved by the Ethics Committee of the Research Institute for Endocrine Sciences. A written informed consent was obtained from each participant.

***Clinical and laboratory measurements***

A pretested-questionnaire was used to collect demographic data, past medical history and family history of CVD, medication use and smoking habits (23). Blood samples were obtained after 12–14 hrs overnight fasting and were centrifuged within 30-45 minutes of collection. Fasting plasma glucose (FPG), TC and HDL-C were assayed. For those who were not on any glucose-lowering medications, the standard 2 hr post challenge plasma glucose (2 hr–PCPG) test was conducted. Blood pressure (BP) was measured twice from the right arm using a standardized mercury sphygmomanometer and the mean of the two measurements was considered as the participant’s BP. Anthropometric measurements including weight and height were also recorded. Weight was measured using a digital scale and recorded to the nearest 100 g. Height was measured in the standing position, using a measuring tape.

***Follow-up and outcome event***

The follow-up period was defined as the period between the initial enrollment in the TLGS study in 1999-2001 until March 20^th^, 2013. Details of the outcome collection have been described previously (24). Details of outcome collection have been described previously (25). In the present study, deaths from CHD were considered as the first event and deaths from stroke were considered as the second event. Death from CHD or stroke was confirmed by reviewing the death certificate or medical records.

## Results

The study sample consisted of 4522 individuals (2502 women and 2020 men,). During the median of 12.4 years of follow-up, 63 deaths from CHD (33 women and 30 men) and 53 deaths from stroke (37 women and 16 men) were registered. Table 1 shows participants’ baseline characteristics and also the mean and standard deviation (SD) or frequency (percentage) of independent covariates. Men were significantly older than women (*P<*0.0001) and had lower SBP (P=0.001) and BMI (*P<*0.0001). Also, the prevalence of diabetes (*P=*0.003) and consuming lipid-lowering medications (*P<*0.0001) were significantly lower in men than in women (Table 1). Men also had lower FBS than women (*P=*0.008). The prevalence of smoking was higher in men (*P=*0.001). 

Table 2 shows the mean and SD and median interquartile range (IQR) of lipid markers at baseline. As the Table shows women had significantly greater TC, LDL-C, and HDL-C than men (*P<*0.0001 for all three variables). There was no significant difference between men and women in TG level (*P=*0.15).

***Cause-specific hazard***

Table 3 shows cause-specific hazard ratios and 95% confidence interval of CHD and stroke events for lipid profile components. Analysis of the association between lipid markers and cause-specific hazard revealed that in women one SD increase in TC or LDL-C level increased the hazard of death by 1.42(CI=1.07,1.89) and 1.41(CI=1.04,1.93), respectively. The impact of one SD increase in TG level on the hazard of CHD death among women was marginally significant (P=0.08). There was no significant association between any of the lipid markers and death due to stroke in women. Furthermore, analysis on the data obtained from men and also sex-adjusted analysis revealed no significant association between lipid markers and hazard of death from CHD and stroke (Table 3).

***Sub-distribution hazard***

Table 4 shows sub-distribution hazard ratios and 95% confidence interval of CHD and stroke events for lipid profile components. Analysis of sub-distribution hazard revealed a significant association between TG level and the cumulative incidence of death from CHD in women; one SD increment in TG level increased the cumulative incidence of death from CHD by 1.94 (CI=1.02,3.75). There was no significant association between the cumulative incidences of death from CHD or stroke with other lipids in either gender and also in the sex-adjusted analysis.

**Figure 1:**
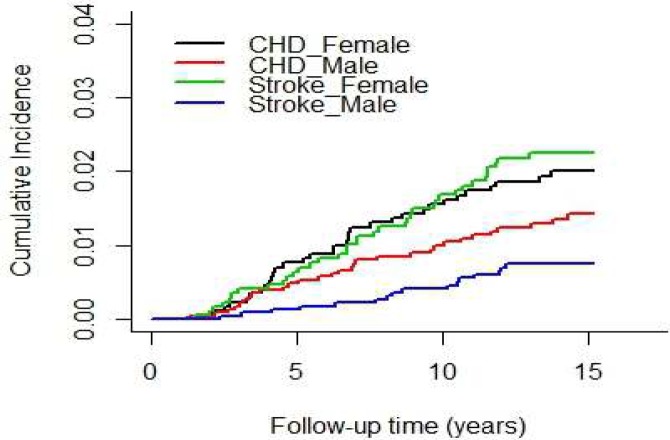
Cumulative incidence curve for each cause of mortality and for each gender, Tehran Lipid and Glucose Study (TLGS) (1999–2012)

Figure 1 shows the cumulative incidence curve for each cause of mortality for men and women separately. As the Figure shows at three years into the follow-up study the cumulative incidence of death from CHD was higher than the cumulative incidence of death from stroke. In addition, the cumulative incidences of death from CHD and stroke were generally greater in women than in men. However, while the cumulative incidence of death due to stroke was significantly higher in women than in men (*P<*0.001), the difference between the two groups in the cumulative incidence of CHD deaths was not significant (*P=*0.137).

**Table 1:** Comparison of baseline characteristics between men and women, Tehran Lipid and Glucose Study (TLGS) (1999–2012)

		**Baseline measurement**	***P*** **-value**
**Age (year)**	Men Women	55.5(9.6) 53.7(8.6)	<0.001
**SBP** **(mm Hg)**	Men Women	126.6(20.6) 128.5(21.1)	0.001
**BMI** **(kg/m )**	Men Women	26.4(3.8) 29.2(4.5)	<0.001
**FBS** **(mg/dL)**	Men Women	104.2(34.4) 107.5(42.1)	0.008
**Smoking(%)**	Men Women	367(22) 51(2.3)	<0.001
**Lipid-lowering medications** **(%)**	Men Women	68(4) 184(8.4)	<0.001
**Diabetes(%)**	Men Women	196(11) 207(14)	0.003

Data are shown as mean (SD) for continuous variables (*P-value* calculated with independent sample t-test), frequency (%) for categorical variables (P-value according to the chi-squared test)

SBP: systolic blood pressure; BMI: body mass index; FBS: fasting blood sugar

**Table 2:** Comparison of lipid markers between men and women, Tehran Lipid and Glucose Study (TLGS) (1999–2012)

		**Mean (SD)**	***P*** **-value** ^a^	**Median (IQR)**	***P*** **-value** ^b^
**TC** **(mg/dL)**	Men Women	215.13(42.61) 235.49(48.72)	<0.001	215(42.64) 235.71(48.66)	<0.001
**LDL-C(mg/dL)**	Men Women	138.46(35.59) 151.48(40.05)	<0.001	138.83(35.71) 151.61(40.03)	<0.001
**HDL-C** **(mg/dL)**	Men Women	38.39(9.37) 44.99(11.25)	<0.001	38.43(9.39) 44.96(11.27)	<0.001
**TG(mg/dL)**	Men Women	200.45(144.42) 198.04(120.82)	0.2	168(120.5) 173(122)	0.15

TC: total cholesterol; HDL-C: high density lipoprotein cholesterol; LDL-C: low density lipoprotein cholesterol; TG: triglyceride

^a^ P value calculated with independent sample t-test

^b^ P value calculated with Mann-Whitney U test

**Table 3:** Cause-specific hazard ratios and 95% confidence interval of CHD and stroke events for lipid profile components, Tehran Lipid and Glucose Study (TLGS) (1999–2012)

		**CHD**		**Stroke**	
		Hazard ratio (95% CI)	***P*** **-value**	Hazard ratio (95% CI)	***P*** **-value**
**Men** ^a^	TC LDL-C TG HDL-C	1.04(0.71,1.54) 1.17(0.8,1.71) 0.82(0.52,1.3) 1.13(0.77,1.65)	0.81 0.4 0.41 0.51	1.005(0.7,1.42) 1.03(0.71,1.48) 1.04(0.73,1.49) 1.1(0.78,1.56)	0.97 0.86 0.8 0.56
**Women** ^a^	TC LDL-C TG HDL-C	1.42(1.07,1.89) 1.41(1.04,1.92) 1.18(0.97,1.44) 1.07(0.75,1.52)	0.01 0.02 0.08 0.69	1.11(0.69,1.77) 1.27(0.78,2.05) 1.1(0.78,1.54) 0.89(0.52,1.42)	0.66 0.32 0.57 0.66
**Overall** ^b^	TC LDL-C TG HDL-C	1.17(0.93,1.46) 1.16(0.92,1.46) 1.09(0.89,1.33) 1.056(0.81,1.37)	0.16 0.19 0.38 0.68	0.89(0.67,1.19) 0.95(0.72,1.27) 1.09(0.85,1.4) 0.87(0.64,1.2)	0.46 0.77 0.47 0.42

TC: total cholesterol; LDL: low density lipoprotein; HDL: high density lipoprotein; TG: triglycerides; P-value based on Cause-specific hazard model

^a ^Model was adjusted for: systolic blood pressure, body mass index, diabetes, smoking, and lipid-lowering medications

^b ^Model was adjusted for: gender, systolic blood pressure, body mass index, diabetes, smoking, and lipid-lowering medications

**Table 4:** Sub-distribution hazard ratio and 95% confidence interval of CHD and stroke events for lipid profile components, Tehran Lipid and Glucose Study (TLGS) (1999–2012)

		**CHD**		**Stroke**	
		Sub-distribution hazard (95% CI)	***P*** **-value**	Sub-distribution hazard (95% CI)	***P*** **-value**
**Men** ^a^	TC LDL-C TG HDL-C	0.6(0.1,3.55) 1.37(0.32,5.85) 0.59(0.31,1.1) 0.95(0. 21,4.18)	0.58 0.66 0.097 0.95	0.69(0.13,3.6) 1.04(0.31,3.44) 0.55(0.26,1.17) 1.09(0.91,4.65)	0.67 0.95 0.12 0.91
**Women** ^a^	TC LDL-C TG HDL-C	3.84(0.553,26.29) 2.28(0.45,11.3) 1.94(1.02,3.75) 0.97(0.16,5.78)	0.17 0.31 0.04 0.98	1.56(0.24,9.78) 1.77(0.33,9.42) 0.93(0.31,2.82) 0.79(0.06,9.75)	0.64 0.5 0.91 0.86
**Overall** ^b^	TC LDL-C TG HDL-C	0.6(0.1,3.55) 1.37(0.32,5.85) 0.96(0.61,1.51) 0.88(0.29,2.65)	0.58 0.66 0.87 0.83	0.44(0.12,1.54) 0.8(0.31,2.02) 0.56(0.3,1.06) 0.56(0.16,1.88)	0.2 0. 64 0.073 0.35

C: total cholesterol; LDL: low density lipoprotein; HDL: high density lipoprotein; TG: triglycerides; *P-value*: based on Sub-distribution hazard (Fine-Gray) model

^a ^Model was adjusted for: systolic blood pressure, body mass index, diabetes, smoking and lipid-lowering medications

^b ^Model was adjusted for: gender, systolic blood pressure, body mass index, diabetes, smoking and lipid-lowering medications

## Discussion

In this study, we investigated the association between TC, LDL-C, HDL-C and triglyceride with hazard of death from CHD and stroke. We also examined the effects of these lipids on cumulative incidence of death from CHD and stroke in a competing risk setting while controlling for potential effects of blood pressure, diabetes, BMI, smoking, age and gender. We observed that only TC and LDL-C increase the hazard of CHD death in women. We also found that only TG increases the cumulative incidence of CHD death in women.

In a previous study on this population, Tohidi and colleagues (7) examined lipid markers’ association with first CHD and stroke (not CHD and stroke mortality) as two competing events in individuals over 50 years of age. In line with the findings of our study, Tohidi *et al*. found no association between the level of any lipid marker and the hazard of stroke in men or women (7). In addition, LDL-C was found a risk factor for CHD in women (7). We found that only TC and LDL-C levels were significantly associated with an increased hazard of CHD mortality in women. However, unlike our study, in the study by Tohidi *et al*. TC and LDL-C were reported as risk factors of CHD in men, while HDL-C was found to be a protective factor for CHD in women (7).

Although TG has been reported as a risk factor of hazard of CHD (5, 6) and stroke (6), in the present study we did not observe any significant association between TG and CHD or stroke mortality in men and women.

Results of the present study showed that TG increases the cumulative incidence of CHD mortality in women. This is in line with findings of the study by Dianatkhah and colleague (26); however, there are studies that do not report a significant association between TG and the cumulative incidence of CHD mortality (19, 20).

LDL-C has been reported as a risk factor of CHD in men (5) and women (16), with no significant association with stroke (5). In the present study, we observed a significant association between LDL-C and CHD in women. This finding is in line with those reported by Everett *et al*. (16). Nevertheless, LDL-C was not associated with stroke. In the PRIME study by Canouï-Poitrine and colleagues (5) no association was reported between stroke and LDL-C.

LDL-C has been previously reported as a risk factor for CVD incidence among Iranian (26). However, we did not observe any association between LDL-C and the cumulative incidence of death from CHD and stroke.

HDL is mostly known as a protective factor for CHD and stroke. The protective effect of HDL-C on CHD has been reported in both men (5) and women (6, 16). The protective effect of HDL-C on stroke has also been reported in both men (16, 18) and women (6, 18). Results of the present study were in line with findings of the study by Canouï-Poitrine* et al*. that showed no significant association between HDL-C and CHD or stroke (5). In addition, no significant association was observed between HDL-C and the cumulative incidence of death from CHD and stroke. Similar results have also been reported in a previous study on Iranian (26).

The present study revealed that TC is a risk factor of CHD in women. This is in agreement with findings of the studies by Iso *et al*. (6) and Everett *et al*. (16). TC was shown as a risk factor of CHD in men (5, 17). Moreover, some studies have shown the protective effect of TC on CHD in men (18) and women (6, 18).

Total cholesterol has been shown to have a significant predictive value for cumulative incidence of CHD (10, 11, 20), and cumulative incidence of CHD death (9), but no significant effect on the cumulative incidence of stroke (20). In a cohort of Iranian with type 2 diabetes, cholesterol had a significant effect on CVD (19). In the present study, we observed no significant association between TC and cumulative incidence of CHD and stroke.

In the present study, we observed that lipid markers are more associated with CHD than with stroke. Similar findings have been observed by Everett *et al*. (16) and Peters *et al*. (27). In addition, lipid markers had greater effect on women than on men.

The current study revealed no association between most lipid markers and the cumulative incidence of death from CHD or stroke. A possible explanation for this finding might be that we considered death from CHD and stroke as two events of interest. It should be noted that most individuals with abnormal level of lipids kept their lipids level under control by using lipid-lowering medications. Additional analyses revealed that 73% and 84% of CHD-related deaths in men and women, respectively, and 100% and 94% of stroke-related deaths in men and women, respectively, occurred in those who did not consume lipid-lowering medications.

The strengths of the present TLGS study includes its population-based prospective design, detailed and systematic follow-up, and the fact that all blood sample measurements were performed at the same center. Our study, however, had some limitations; it was conducted among Persian ethnicities, and hence the results cannot be generalized to other populations. In addition, the number of events was too small (especially in men - 16 strokes), and this yielded a loss of power in detecting significant associations. Furthermore, we applied a modified Friedewald formula to calculate the level of LDL-C rather than measuring it directly. In addition, this study did not control for other risk factors of CVD, such as lack of physical activity, unhealthy diet, stress and family history.

## Conclusion

The associations of lipid components on CHD death were modiﬁed by gender. TC, LDL-C and TG were independent predictors of CHD mortality in women. Furthermore, death due to stroke changes the association of lipid markers with CHD mortality. This study highlights the importance of considering competing risks analysis and reporting the results of both cause-specific and sub-distribution hazard analyses.

## Conflicts of Interest

The authors declare that there are no conflicts of interest. 
